# Materials and Bioactive Factors in Dental Restoration and Periodontal Therapy

**DOI:** 10.1155/2016/4809051

**Published:** 2016-02-09

**Authors:** Vesna Miletic, Tihana Divnic-Resnik, Natasa Nikolic Jakoba, Andrija Petar Bosnjak, Paulo Henrique Perlatti D'Alpino

**Affiliations:** ^1^DentalNet Research Group, School of Dental Medicine, University of Belgrade, 11000 Belgrade, Serbia; ^2^Discipline of Periodontics, Faculty of Dentistry, The University of Sydney, Sydney, NSW 2010, Australia; ^3^Department of Periodontology and Oral Medicine, School of Dental Medicine, University of Belgrade, 11000 Belgrade, Serbia; ^4^School of Medicine, University Josip Juraj Strossmayer, 31000 Osijek, Croatia; ^5^School of Medicine, University of Rijeka, 51000 Rijeka, Croatia; ^6^School of Dentistry, Biomaterials Research Group, Universidade Anhanguera de São Paulo, 05145-200 São Paulo, SP, Brazil

Dental restoration and periodontal therapy have undergone tremendous expansion over the past few decades. Improvements have been made in basic and applied material science but also in clinical considerations of functional, esthetic, reparative, and regenerative aspects of materials and bioactive factors. This special issue offers a wide range of topics, broadening knowledge of researchers, dental specialists, and general dental practitioners.

Dentin-adhesive bond remains a challenge in modern adhesive dentistry due to the complex composition and morphology of dentin. Different treatment modalities have been proposed to improve the dentin-adhesive bond and increase its longevity. Among these, conditioning dentin with ethylenediaminetetraacetic acid (EDTA) along with carbodiimide pretreatment have shown potential in preserving bond strength to dentin of self-etch adhesives over time.

Impression materials should withstand tear and tensile forces and be able to recover fully for an ideal impression of intricate details of dental and oral structures. A number of hydrophilic elastomeric impression materials are available on the market. In vitro testing of tensile properties of heavy-body, medium-body, and light-body polyvinylsiloxane, polyether, and vinylpolyether silicone commercial products offers scientific data for clinical selection of impression materials for specific applications.

Platelet-rich fibrin membranes seem to improve the healing process in periodontal regenerative treatments. Leukocyte- and platelet-rich fibrin (L-PRF) is considered a second-generation platelet concentrate, able to form strong fibrin matrices. Early L-PRF membranes have shown stronger mechanical properties, namely, tensile strength, modulus of elasticity, and toughness, than membranes obtained using platelet rich in growth factors (PRGF)/Endoret® technology, indicating potential clinical advantages.

Titanium and titanium alloys are nowadays considered the material of choice for dental implant applications in the replacement of missing teeth. Numerous studies focus on the mechanisms of interaction between the surface of titanium or titanium alloys and host tissues. Recently, the role of titanium oxide surface layer in osteoblast differentiation has been studied in terms of alkaline phosphatase (ALP) activity. Increased ALP activity suggests that titanium oxide acts as a bioactive factor involved in osteoblast differentiation and subsequent osseointegration.

Finite element analysis (FEA) has been used in assessment of stress distribution induced by occlusal loading in diverse dental tissues, restorations, implants, and surrounding alveolar bone (cancellous and cortical). FEA may be particularly useful in designing dental implants. Extensive numerical simulation reveals complex stress distribution in and around dental implants with different thread designs and abutment angulations restored with porcelain crowns and subjected to a number of occlusal loading conditions.

*Vesna Miletic* *Vesna Miletic* *Tihana Divnic-Resnik* *Tihana Divnic-Resnik* *Natasa Nikolic Jakoba* *Natasa Nikolic Jakoba* *Andrija Petar Bosnjak* *Andrija Petar Bosnjak* *Paulo Henrique Perlatti D'Alpino* *Paulo Henrique Perlatti D'Alpino*

